# Effect of body position on ventilation distribution during PEEP titration in a porcine model of acute lung injury using advanced respiratory monitoring and electrical impedance tomography

**DOI:** 10.1186/s40635-014-0038-6

**Published:** 2015-01-31

**Authors:** Klaus Pfurtscheller, Stefan Ring, Elisabeth Beran, Erich Sorantin, Joachim Zobel, David Ganster, Alexander Avian, Gerfried Zobel

**Affiliations:** Pediatric Intensive Care Unit, University Children’s Hospital and Medical University Graz, Auenbruggerplatz 34, 8036 Graz, Austria; Division of Cardiac Surgery, Department of Surgery, Medical University Graz, Auenbruggerplatz 29, 8036 Graz, Austria; Division of Pediatric Radiology, Department of Radiology, Medical University Graz, Auenbruggerplatz 34, 8036 Graz, Austria; Institute for Medical Informatics, Statistics and Documentation, Medical University Graz, Auenbruggerplatz 2, 8036 Graz, Austria

**Keywords:** Acute lung injury, Prone position, Positive end expiratory pressure, Volumetric capnography, Electrical impedance tomography, Alveolar dead space

## Abstract

**Background:**

Lung failure after acute lung injury remains a challenge in different clinical settings. Various interventions for restoration of gas exchange have been investigated. Recruitment of collapsed alveoli by positive end expiratory pressure (PEEP) titration and optimization of ventilation-perfusion ratio by prone positioning have been extensively described in animal and clinical trials. This animal study was conducted to investigate the effects of PEEP and positioning by means of advanced respiratory monitoring including gas exchange, respiratory mechanics, volumetric capnography and electrical impedance tomography.

**Methods:**

After induction of acute lung injury by oleic acid and lung lavage, 12 domestic pigs were studied in randomly assigned supine or prone position during a PEEP titration trial with maximal PEEP of 30 mbar.

**Results:**

Induction of lung injury resulted in significant deterioration of oxygenation [partial pressure of arterial oxygen/inspiratory fraction of oxygen (PaO_2_/FiO_2_): *p* = 0.002] and ventilation [partial pressure of arterial carbon dioxide (PaCO_2_): *p* = 0.002] and elevated alveolar dead-space ratios (Valv/Vte: *p* = 0.003) in both groups. Differences in the prone and the supine group were significant for PaCO_2_ at incremental PEEP 10 and 20 and at decremental PEEP 20 (20d) and 10 (10d), for PaO_2_/FiO_2_ at PEEP 10 and 10d and for alveolar dead space at PEEP 10d. Electrical impedance tomography revealed homogenous ventilation distribution in prone position during PEEP 20, 30 and 20d.

**Conclusions:**

Prone position leads to improved oxygenation and ventilation parameters in a lung injury model. Respiratory monitoring with measurement of alveolar dead space and electrical impedance tomography may visualize optimized ventilation in a PEEP titration trial.

## Background

Mortality of the acute respiratory distress syndrome (ARDS), first described in 1967 by Ashbough et al. [1], remained constant over three decades in adults and children despite extensive clinical and experimental research efforts [2-6]. Pathophysiologic alterations of ARDS lead to extensive atelectasis which might partly be re-opened and therefore re-ventilated. On the other hand, regions of consolidation might develop which cannot be ventilated in the acute phase [7,8]. This process leads to a considerable reduction of end-expiratory lung volume with a reduced surface for gas exchange and therefore results in hypoxemia. Furthermore, there are big differences within the regional respiratory mechanics of the lung [8]. Visualization of regions of alveolar collapse or hyperinflation is possible with the help of computed tomography (CT) scans [9-12] or lung scintigraphy [13]. However, these techniques are not bedside methods and therefore have a limited applicability in sick intensive care patients and children. The electrical impedance tomography (EIT) is a non-invasive method for the evaluation of the global and regional ventilation state. EIT is based on the differences of electric conductivity of tissue with different or changing impedance. The (spatial) resolution of EIT is much lower than that of CT and therefore cannot be used for the exact representation of lung morphology. However, experimental and clinical data showed a good correlation between EIT and CT in terms of regional distribution of ventilation [14-22].

The introduction of lung-protective ventilation with a tidal volume (TV) of 6 ml/kg idealized body weight significantly reduced the incidence of ventilator-induced lung injury (VILI) in patients with ARDS [23]. Whereas a reduced TV is the gold standard for lung-protective ventilation, there exist no distinct recommendations concerning the ideal positive end expiratory pressure (PEEP). Different methods for the setting of PEEP have been described: a fixed combination of inspiratory fraction of oxygen (FiO_2_) and PEEP, adjustment according to the pressure-volume curve or to the maximal partial pressure of arterial oxygen (PaO_2_) and the minimal partial pressure of arterial carbon dioxide (PaCO_2_) and adjustment after a CT scan [24-30]. However, the ideal PEEP has to be found in particular for any individual patient. By means of volumetric capnography (VC), the elimination of CO_2_ (carbon dioxide) with each breath is measured and dead-space volumes can be illustrated and calculated. By increasing inspiratory and expiratory lung volume, a certain point with maximal CO_2_ elimination per breath can be reached. If the lung volume is changed from this point on, the CO_2_ elimination will be reduced. Therefore, the optimal lung volume can be found with the help of volumetric capnography [29]. A further approach to improve ventilation of the dorsal lung areas is the prone position. Numerous studies have demonstrated an improvement of oxygenation without having an influence on the course of ARDS [31-38] whereas recent studies also show better middle-term survival rates [39]. Prone position results in improved ventilation of dorsal lung regions and a more homogenous ventilation and perfusion of altered lung parenchyma.

The aim of this study was to evaluate non-invasive parameters for global and regional ventilation and CO_2_ elimination in a porcine model of acute lung injury (ALI) in supine and prone position using a PEEP titration protocol.

Study objectives in this porcine ALI model were as follows:

- Change of dead-space fractions [airway dead space (Vdaw), physiologic dead space (Vdphys), alveolar dead space (Vdalv)] during PEEP titration in prone and supine position.
- Non-invasive monitoring of distribution of aerated and non-aerated lung regions by means of EIT in prone and supine position.

## Methods

The protocol was approved by the Institutional Animal Research Committee (Reference number BMWF-66010/0004-II/3b/2012), and the experiments were performed in accordance with European and Austrian laws on animal experimentation and with the guidelines for ethical animal research. The animals were hosted at the Division of Biomedical Research (BBF), Medical University of Graz, Austria. Veterinary medical experts performed anaesthesia and supervised the study period according to the protocol of the BBF. The Division works according to FELASA and GV-SOLAS guidelines and was certified by ISO 9001 in 2008.

### Animal preparation

Twelve domestic female pigs, weighing 35.7 ± 3.9 kg (mean ± SD) were included in the study after acclimatization for several days at our animal care institution. Conventional pig husbandry in designated large animal rooms was carried out in enclosures that are appropriate for the respective species. After intramuscular premedication with midazolam (0.5 mg/kg), ketanest (10 mg/kg) and atropine (0.1 mg/kg) and cannulation of an auricular vein, anaesthesia was induced with a propofol injection. Thereafter anaesthesia was maintained by a continuous infusion with propofol (8 mg/kg/h), ketanest (10 mg/kg/h), pancuronium (0.3 mg/kg/h) and fentanyl (3.5 μg/kg/h) with little dosage adaptations according to individual purposes.

After placement in supine position and orotracheal intubation (UnoFlex™ spiral tube, HVLP cuff, Magill, 8.0 to 8.5 mm I.D.; pfm medical, Cologne, Germany), controlled mechanical ventilation was established. Initial ventilator (Draeger Evita Infinity® V 500; Draeger medical, Luebeck, Germany) settings within a volume-controlled mode were tidal volume (Vt) 8 ml/kg, PEEP 5 cm H_2_O, respiration rate (RR) 20 breaths/min, I:E = 1:2 and FiO_2_ 0.3 according to the protocol aiming to a PaCO_2_ of 35 to 40 Torr (PaCO_2_ mean 38.6 ± 4.4 Torr). Continuous fluid replacement was provided by 0.9% saline and 5% glucose with a rate of 5 ml/kg/h each, with adjustments targeting a central venous pressure of 10 mmHg.

### Invasive monitoring

For the invasive monitoring, the cervical vessels on both sides were surgically prepared. A 4 French (Fr) arterial thermodilution catheter (PICCO, Pulsion Medical Systems SE, Feldkirchen, Germany) was inserted in the internal carotid artery and a 4.5 Fr venous oxymetric catheter (Edwards Lifesciences Corp, Irvine, USA) was placed in the internal jugular vein. Furthermore, a second arterial line (20 gauge, Abbocath) and a second central venous catheter (5 Fr, 3-lumen) were established on the other side. A 20-gauge catheter (BD™ arterial line with Floswitch™, Becton Dickinson, Franklin Lakes, USA) was placed in one femoral artery for drawing the arterial blood probes. After these surgical applications, we administered unfractioned heparin (100 IU/kg) intravenously. Transabdominal catheterism of the urinary bladder was performed for collection of urine.

### Non-invasive monitoring

Non-invasive monitoring consisted of transthoracic ECG and peripheral pulse oximetry (SpO_2_) from the tip of the tail. The central temperature was measured using an esophageal temperature probe.

### Hemodynamic monitoring

Heart rate (HR) and rhythm, arterial blood pressures [systolic (RRsyst), diastolic (RRdiast) and mean (RRmean)] and central venous pressure (CVP) were recorded continuously, whereas all pressures were referenced to the mid-thorax. Central venous saturation (SsvcO_2_) and cardiac parameters (CO, CI) were recorded, but results are not available for statistical analysis due to incomplete data generation.

### Respiratory monitoring

Respiratory parameters [respiratory rate (RR), inspiratory and expiratory tidal volumes (Vti, Vte), airway pressures - peak inspiratory pressure (PIP), plateau pressure (Pplat), mean airway pressure (Paw), PEEP, dynamic compliance (Cdyn), expiratory airway resistance (Rawexsp)] and the end-tidal partial pressure of carbon dioxide (etCO_2_) were measured with the Draeger Infinity etCO_2_ + Respiratory Mechanics Pod® using the Capnostat III® infrared mainstream sensor placed at the airway opening combined with an adult/paediatric flow sensor. A zero calibration was performed before starting the measurement phase of the protocol. Cdyn was measured during uninterrupted mechanical ventilation and calculated as the expiratory tidal lung volume divided by the ventilatory pressure amplitude [VTexp/(PIP − PEEP)]. All parameters were continually recorded using the Draeger Infinity Delta Series (Draeger medical GmbH, Luebeck, Germany). Arterial and central venous blood samples were taken for measurements of haemoglobin (Hb), oxygen saturation (SaO_2_, SvO_2_), arterial and venous PO_2_ (PaO_2_, PvO_2_) and PCO_2_ (PaCO_2_, PvCO_2_), pH (a-pH, v-pH) and lactate (a-lact, v-lact) using an automatic blood gas system (IL GEM® Premier 3000; Instrumentation Laboratory GmbH, Kirchheim, Germany).

### Volumetric capnography (VC) and dead-space fractions

The etCO_2_ + Respiratory Mechanics Pod® displayed etCO_2_, VCO_2_ (CO_2_ minute ventilation), PECO_2_ (mixed partial pressure of CO_2_ of a single expiration), airway dead-space fraction (Vdaw/Vte) and respiratory mechanics. Tidal elimination of CO_2_ (VtCO_2_) was calculated by integrating the product of flow and CO_2_ concentration (area under the CO_2_ curve) measured by the mainstream infrared sensor during expiratory flow. Physiologic dead-space fraction was calculated according to the Bohr-Enghoff formula Vdphys/Vte = (PaCO_2_ − PECO_2_)/PaCO_2_. Alveolar dead-space fraction (Vdalv/Vte) was calculated as the difference between physiologic dead-space fraction and airway dead-space fraction. [physiologic dead space (Vdphys/Vte), airway dead space (Vdaw/Vte), alveolar dead space (Vdalv = Vdphys − Vdaw)]. For an overview of respiratory dead-space measurements, we refer to Tang et al. [40].

For measuring global and regional ventilation, an EIT system (Pulmovista 500®, Draeger medical GmbH, Luebeck, Germany) was installed with continuous and automated data collection. For fixing the electrodes, the smallest available electrode belt with regular electrode positions around the thorax fixed caudally of the axillary folds was used. For a comprehensive review on the principles of EIT, we refer to Bodenstein et al. [41].

### Experimental protocol

After installation of the complete monitoring, the animals were allowed to rest in supine position for 30 min before the baseline measurement (measurement point: BASELINE). Then, the experimental lung injury was induced by slow intravenous injection of oleic acid (0.09 ml/kg) over about 1 h (time of injection: 65 ± 25 min; mean ± SD). Thereafter, an endotracheal lung lavage was performed by instilling and draining warmed 0.9% saline with amounts of up to 30 ml/kg until ALI was established which was defined as partial pressure of arterial oxygen/inspiratory fraction of oxygen (PaO_2_/FiO_2_) < 100 Torr for 30 min (MPALI1 - not represented in graphs and tables). This combined technique of intravenous oleic acid infusion and lung lavage with 0.9% saline has proven to induce a stable and severe ALI in different animal species resulting from an inflammatory response associated with a pulmonary capillary leak and from pulmonary surfactant deficiency [42]. Blood pressure reactions during oleic acid injection and lung lavage were compensated with norepinephrine (max 0.1 μg/kg/min) or phenylephrine (max 0.1 μg/kg/min). During this procedure, ventilation parameters were adapted (volume-controlled ventilation, Vt 6 ml/kg, PEEP 5 cm H_2_O, respiration rate 20 to 24 breaths/min, I:E = 1:2 and FiO_2_ 1.0). Then, the pigs were randomized to the supine position (SP, *n* = 6) or the prone position (PP, *n* = 6) group [The first pig acutely died after induction of ALI before the trial. Therefore, one pig (pig 11) had to be measured in both positions (SP and PP) with an adequate time-interval in-between to avoid an advantage of the precedent lung recruitment].

**Figure 1:**
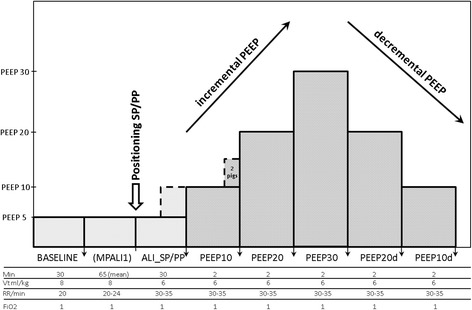
**Schema of study protocol.** Twelve animals were studied with measurement points at BASELINE, after positioning in supine or prone position (ALI_SP/PP) and at different incremental (PEEP 10, 20, 30) and decremental PEEP levels (PEEP 20d, 10d). Time frame and ventilator settings are illustrated on the *x*-axis.

After randomization and positioning (measurement point: ALI_SP/PP), the PEEP titration was performed according to the protocol in the volume-controlled mode without upper peak pressure limit (ventilator settings: Vt 6 ml/kg, RR 30 to 35 breaths/min, I:E = 1:2 and FiO_2_ 1.0). PEEP titration was started at a PEEP of 10 mbar (two pigs in the SP group clinically required a minimal PEEP of 15 mbar at this time point but were still included in the study) with a stepwise increase of 5 mbar until a plateau PEEP of 30 mbar followed by a stepwise reduction of the PEEP to a minimum of 5 mbar (0 mbar when tolerated). PEEP changes were done every 2 min, and complete data with blood drawings were collected after each 10-mbar PEEP change (measurement points: PEEP 10, 20, 30, 20d, 10d) (Figure 1).

### Data analysis

Automatically collected data from the Draeger Infinity Delta system were visually viewed for consistent time periods of 1 min per measurement point (BASELINE − ALI_SP/PP − PEEP 10 − PEEP 20 − PEEP 30 − PEEP 20d − PEEP 10d). Sixty values of these 1-min time periods (1 value per second) were averaged and used for further analysis. EIT data which were displayed on the Pulmovista 500® were documented at each measurement point.

Values are given as medians with interquartiles (25^th^ and 75^th^) since assumptions of normality were not always satisfied. For group comparisons the Mann-Whitney *U*-Test and for the analysis of changes between measurement points within each group the Friedman test followed by Wilcoxon's signed rank test were performed. A *p* value of <0.05 was considered statistically significant.

## Results

### Global physiologic and respiratory variables

**Figure 2:**
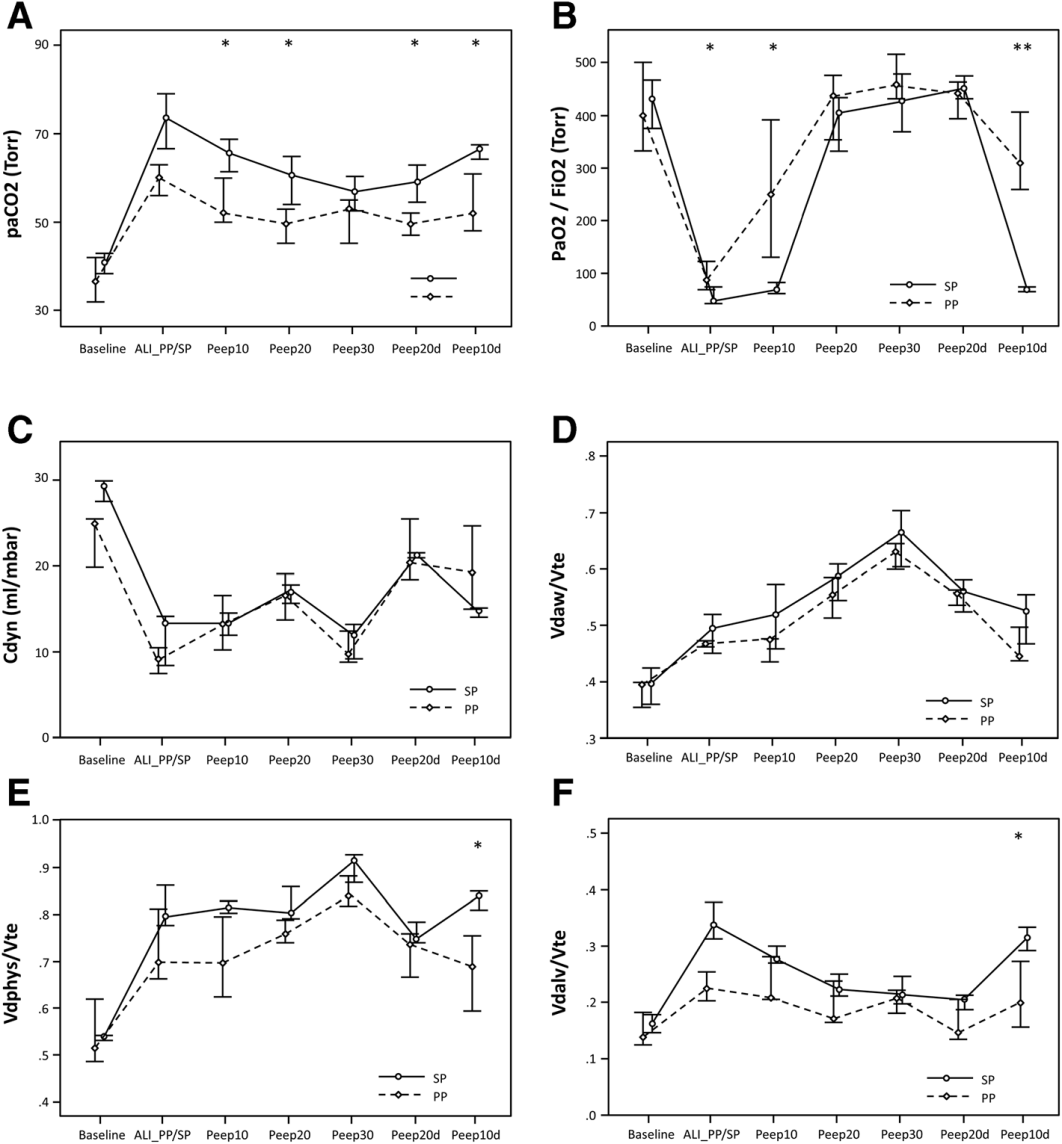
**Advanced respiratory monitoring during the PEEP trial in a porcine ALI model.** Illustrated are the changes of paCO_2_ **(A)** and PaO_2_/FiO_2_ **(B)** reflecting ventilation and oxygenation, respectively, and dynamic lung compliance, Cdyn **(C)**. Dead space (DS) is presented as fractions of expiratory tidal volume; illustrated are airway DS **(D)**, physiologic DS **(E)** and alveolar DS **(F)**. Measurement points are before ALI induction at BASELINE, after randomly positioning in either supine or prone position at ALI_SP/PP; at incremental PEEP 10, 20 and 30; and at decremental PEEP 20d and 10d. Full lines represent supine and broken lines prone position. Values are medians and interquartile range (IQR); *significant group differences *p* < 0.05; **significant group differences *p* < 0.01.

Cardiocirculatory (HR, MAP) and respiratory (RR, PIP, PAW, Vte) variables reacted similarly in the supine (*n* = 6) and the prone groups (*n* = 6) at BASELINE, ALI_SP/PP and within the PEEP trial, reflecting the stability of the injury model within the experimental trial (Table 1). PaCO_2_ increased significantly after ALI induction [PP: BASELINE: 36.5 and ALI_PP: 60.0; SP: BASELINE: 39.5 and ALI_SP: 73.5 (Torr; medians), *p* = 0.002 for both groups] and was significantly different between the groups at PEEP 10 (PP: 52.0; SP: 65.5), PEEP 20 (PP: 49.5; SP: 65.0), PEEP 20d (PP: 49.5; SP: 59.0) and PEEP 10d [PP: 52.0; SP: 67.5 (each Torr, medians), *p* < 0.05] with overall lower values in the prone group (Figure 2A). After proning, etCO_2_ remained without significant differences between the groups except at PEEP 20 [PP: 46.2; SP: 51.5 (Torr, medians), *p* < 0.05]. The PaO_2_/FiO_2_ ratio reflecting the trans-pulmonary oxygen transport showed a significant decline after induction of ALI in both groups [PP: BASELINE: 400.0 and ALI_PP: 88.5; SP: BASELINE: 375.0 and ALI_SP: 48.5 (Torr; medians), *p* = 0.002 for both groups]. During the trial, there were significant differences of the PaO_2_/FiO_2_ ratio between groups at ALI_SP/PP [PP: 88.5; SP: 48.5 (Torr, medians), *p* < 0.05], PEEP 10 [PP: 248.5; SP: 69.0 (Torr, medians), *p* < 0.05] and PEEP 10d [PP: 310.0; SP: 64.5 (Torr, medians), *p* < 0.01] (Figure 2B). Compliance (Cdyn) showed a similar behaviour during the PEEP trial within the two groups with no significant differences. After a significant decline of Cdyn of both groups from baseline to ALI [PP: BASELINE: 24.9 and ALI_PP: 9.05; SP: BASELINE: 28.4 and ALI_SP: 13.35 (ml/mbar; medians), *p* = 0.002 for both groups], the PEEP trial resulted in an increase of Cdyn until PEEP 20 with a successive decline during PEEP 30 reflecting overdistension in both groups. Decremental PEEP resulted in an elevation of Cdyn during PEEP 20d. At PEEP 10d, Cdyn was insignificantly higher in the prone group [PP: PEEP 10d: 19.4; SP: PEEP 10d: 14.5 (ml/mbar; medians), *p* = 0.132] (Figure 2C).

**Table 1:** **Hemodynamic and ventilatory variables, data of gas exchange, dead space and EIT during PEEP trial**

		**BASELINE**	**ALI_PP/SP**	**PEEP 10**	**PEEP 20**	**PEEP 30**	**PEEP 20d**	**PEEP 10d**
HR, beats/min	SP	93 (76 to 122)	135 (130 to 145)	118 (115 to 124)	124 (122 to 139)	153 (124 to 160)	143 (137 to 150)	124 (122 to 157)
	PP	92 (90 to 106)	117 (116 to 129)	121 (110 to 147)	126 (120 to 157)	140 (135 to 184)	146 (128 to 192)	129 (109 to 184)
MAP, mmHg	SP	80 (67 to 84)	101 (85 to 113)	76 (32 to 108)	89 (79 to 107)	78 (67 to 82)	96 (90 to 110)	107 (95 to 121)
	PP	95 (72 to 107)	81 (63 to 89)	87 (77 to 99)	72 (60 to 83)	62 (55 to 67)	85 (70 to 95)	100 (90 to 108)
RR, breaths/min	SP	26 (26 to 28)	31 (30 to 34)	34 (34 to 35)	34 (34 to 35)	34 (34 to 35)	34 (34 to 35)	34 (34 to 35)
	PP	26 (26 to 28)	33 (26 to 34)	33 (30 to 34)	33 (30 to 34)	33 (30 to 34)	33 (30 to 34)	33 (30 to 34)
PIP, cm H_2_O	SP	16 (14 to 19)	28 (26 to 29)	28 (26 to 28)	34 (34 to 35)	49 (47 to 52)	30 (30 to 31)	25 (24 to 27)
	PP	19 (17 to 20)	30 (25 to 32)	28 (24 to 32)	34 (32 to 37)	52 (51 to 57)	31 (30 to 33)	21 (19 to 29)
PAW, mbar	SP	9 (8.3 to 9)	16 (14 to 17)	16 (16 to 17)	26 (25 to 26)	37 (36 to 38)	24 (24 to 25)	16 (16 to 18)
	PP	9 (9.1 to 9.6)	14 (13 to 15)	17 (15 to 19)	26 (25 to 27)	39 (37 to 41)	24 (24 to 25)	14 (14 to 18)
Vte, ml	SP	286 (277 to 360)	212 (191 to 238)	201 (191 to 211)	202 (183 to 239)	203 (182 to 234)	201 (180 to 235)	201 (183 to 235)
	PP	279 (275 to 324)	213 (197 to 256)	207 (187 to 251)	212 (184 to 254)	208 (192 to 233)	207 (190 to 249)	209 (190 to 251)
PaCO2, Torr	SP	39 (36 to 42)	73 (62 to 82)	65 (61 to 68)*	65 (56 to 71)*	60 (55 to 69)	59 (56 to 64)*	67 (66 to 79)*
	PP	36 (35 to 42)	60 (56 to 63)	52 (50 to 60)	49 (45 to 53)	53 (45 to 55)	49 (47 to 52)	52 (48 to 61)
etCO2, Torr	SP	40 (37 to 44)	43 (39 to 51)	49 (46 to 52)	51 (49 to 59)*	55 (50 to 60)	57 (52 to 63)	43 (39 to 50)
	PP	40 (37 to 44)	49 (46 to 52)	46 (46 to 48)	46 (44 to 49)	49 (46 to 53)	50 (47 to 56)	47 (41 to 50)
PaO_2_/FiO_2_	SP	375 (333 to 450)	48 (43 to 52)*	69 (61 to 82)*	332 (252 to 434)	427 (387 to 445)	433 (348 to 467)	64 (47 to 72)**
	PP	400 (333 to 500)	88 (69 to 122)	248 (131 to 390)	438 (353 to 476)	458 (431 to 516)	442 (393 to 463)	310 (259 to 406)
Cdyn, ml/mbar	SP	28.4 (25.6 to 29.8)	13.3 (8.4 to 14.2)	13.4 (11.9 to 14.6)	17.4 (15.7 to 18.9)	12.6 (9.2 to 13.5)	21.4 (20.9 to 25.1)	14.5 (13.6 to 15.1)
	PP	24.9 (19.8 to 25.5)	9.0 (7.5 to 10.5)	13.2 (10.2 to 16.6)	16.6 (13.7 to 19.1)	9.8 (8.8 to 12.4)	20.5 (18.4 to 25.5)	19.4 (15.0 to 24.7)
Vdaw/Vte	SP	0.40 (0.39 to 0.41)	0.49 (0.47 to 0.54)	0.54 (0.50 to 0.55)	0.59 (0.59 to 0.62)	0.65 (0.63 to 0.67)	0.56 (0.55 to 0.57)	0.52 (0.44 to 0.54)
	PP	0.40 (0.35 to 0.42)	0.47 (0.46 to 0.50)	0.47 (0.43 to 0.51)	0.57 (0.51 to 0.60)	0.63 (0.60 to 0.64)	0.56 (0.53 to 0.59)	0.47 (0.44 to 0.51)
Vdalv/Vte	SP	0.16 (0.14 to 0.17)	0.31 (0.29 to 0.34)	0.28 (0.27 to 0.29)	0.23 (0.22 to 0.24)	0.23 (0.20 to 0.24)	0.20 (0.17 to 0.21)	0.30 (0.28 to 0.33)*
	PP	0.13 (0.12 to 0.18)	0.24 (0.20 to 0.26)	0.23 (0.20 to 0.28)	0.19 (0.16 to 0.24)	0.21 (0.18 to 0.22)	0.14 (0.11 to 0.20)	0.20 (0.15 to 0.27)
Vdphys/Vte	SP	0.54 (0.53 to 0.58)	0.79 (0.76 to 0.86)	0.82 (0.80 to 0.83)	0.81 (0.79 to 0.86)	0.89 (0.87 to 0.93)	0.74 (0.70 to 0.78)	0.83 (0.75 to 0.85)*
	PP	0.51 (0.49 to 0.62)	0.70 (0.66 to 0.81)	0.70 (0.63 to 0.80)	0.76 (0.74 to 0.79)	0.84 (0.82 to 0.88)	0.74 (0.67 to 0.76)	0.69 (0.59 to 0.75)
ROI 1–2, non-dep	SP	24.0 (23.0 to 25.0)	28.5 (24.5 to 32.5)	25.5 (24.5 to 30.0)	18.0 (16.0 to 21.0)***	21.5 (19.5 to 22.5)***	18.8 (17.0 to 21.0)***	25.0 (24.0 to 27.5)
ROI 3–4, dep	SP	23.8 (22.5 to 25.5)	20.0 (15.5 to 24.0)	22.5 (18.0 to 24.0)	30.5 (28.0 to 33.0)	28.0 (27.0 to 29.0)	30.8 (29.0 to 32.5)	23.5 (21.5 to 24.5)
ROI 3–4, non-dep	PP	22.5 (21.5 to 23.5)	39.0 (30.0 to 40.5)***	35.8 (32.5 to 37.0)***	26.3 (24.0 to 27.0)	24.8 (23.5 to 26.5)	23.3 (23.0 to 24.5)	32.3 (29.5 to 33.5)***
ROI 1–2, dep	PP	25.0 (24.0 to 26.5)	10.0 (8.5 to 19.0)	13.0 (11.5 to 16.5)	22.5 (22.0 to 25.0)	24.3 (22.5 to 25.0)	26.0 (24.5 to 26.0)	16.5 (16.0 to 19.5)

HR, heart rate; MAP, mean arterial pressure; RR, respiratory rate; PIP, peak inspiratory pressure; PAW, mean airway pressure; Vte, expiratory tidal volume; PaCO_2_: partial pressure of arterial carbon dioxide; etCO_2_, end-tidal partial pressure of carbon dioxide; PaO_2_/FiO_2_, partial pressure of arterial oxygen/inspiratory fraction of oxygen; Cdyn, dynamic compliance; Vdaw/Vte, airway dead-space fraction; Vdalv/Vte, alveolar dead-space fraction; Vdphys/Vte, physiologic dead-space fraction; ROI, region of interest; non-dep, non-dependent; dep, dependent. Values are medians and interquartile range (IQR). *Significant group differences *p* < 0.05; **significant group differences *p* < 0.01; ***significant inner-group differences *p* < 0.05.

### Dead-space variables

Dead-space variables are illustrated as fractions of expiratory tidal volume. Induction of ALI resulted in increased dead-space variables independent of the positioning [Vdalv/Vte: PP: BASELINE: 0.13 and ALI_PP: 0.24; SP: BASELINE: 0.16 and ALI_SP: 0.31 (medians), *p* = 0.003 for both groups]. Vdaw and Vdphys increased with incremental PEEP and decreased again with decremental PEEP (Figure 2D,E). At PEEP 10d, the prone group had significantly lower values of Vdalv/Vte [PP: 0.2; SP: 0.3; (median), *p* < 0.05] and Vdphys/Vte [PP: 0.69; SP: 0.83; (median), *p* < 0.05] than the supine group. The PP group had overall lower values within the whole experimental protocol (Figure 2E,F; Table 1).

### EIT

**Figure 3:**
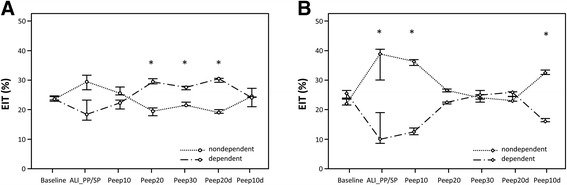
**Electrical impedance tomography reflecting regional ventilation distribution of dependent and non-dependent lung regions during PEEP trial.** Illustrated are the supine group in **(A)** and prone group in **(B)**. Dotted lines represent the non-dependent and broken lines the dependent lung regions. In **(A)**, significant inner-group differences between non-dependent and dependent regions are marked by an asterisk at PEEP 20, 30 and 20d reflecting inhomogenous ventilation distribution. In the prone group **(B)**, ventilation distribution is significantly different within the group at ALI_SP/PP and PEEP 10 and 10d, whereas it is homogenized at PEEP 20, 30 and 20d. *Significant inner-group differences *p* < 0.05.

EIT data were further analysed offline for the supine and the prone group. According to the regular Pulmovista 500® data display, global tidal variation was automatically divided into four symmetrical non-overlapping quadrants of equal size resulting in two non-dependent [SP: region of interest (ROI) 1-2, PP: ROI 3-4] and two dependent (SP: ROI 3-4, PP: ROI 1-2) ROIs in each group. The values of the ROIs represented the percentage of the regional tidal variation of total impedance change. The resulting values of the two ROIs of dependent and non-dependent regions, respectively, were added and averaged for analysis and image presentation. At baseline, both groups showed homogenous ventilation distribution between non-dependent and dependent regions. In the supine group, induction of ALI reduced the ventilation in the dependent areas [ALI_SP: SP_ROI 1-2: 28.5; SP_ROI 3-4: 20.0; (%, medians)] whereas during incremental PEEP, the dependent areas of the lungs are better aerated compared to the non-dependent areas, reflecting inhomogeneous ventilation distribution based on overdistension of the non-dependent areas and recruitment of collapsed areas in the dependent regions. At PEEP 10d, ventilation is again homogeneously distributed (Figure 3A and Table 1). In the PP group, ventilation is very inhomogeneous after the proning manoeuvre with significantly more aeration in the dorsal thus non-dependent regions at ALI_PP [PP_ROI 3-4: 39.0; PP_ROI 1-2: 10.0 (%, medians), *p* < 0.05] and PEEP 10 [PP_ROI 3-4: 35.8; PP_ROI 1-2: 13.0 (%, medians), *p* < 0.05]. At PEEP 20, PEEP 30 and PEEP 20d, ventilation distribution is very homogeneous between dependent and non-dependent lung regions in the prone group (Figure 3B).

## Discussion

Our main findings are a) ventilation and CO_2_-elimination are significantly improved in the prone position; b) by means of EIT, the homogenous distribution of ventilation can be visualized; and c) Cdyn and alveolar dead-space fraction (Vdalv) help to define the optimal PEEP in ALI.

Turning a subject from supine to prone position brings increased chest wall rigidity and decompression of dorsal lung regions, both resulting in a better gas-to-tissue ratio in the dorsal thus non-dependent lung regions [36]. Therefore, gas exchange is ameliorated due to a more homogeneous ventilation distribution and decreased ventilation-perfusion inequalities. Because of evened trans-pulmonary forces, stress and strain are more homogenously distributed and result in reduced VILI [43]. Positive effects of proning on oxygenation in ARDS are confirmed by recent meta-analyses in adult humans [44,45]. Our results demonstrated that immediately after the proning manoeuvre, oxygenation improved significantly in the prone group whereas arterial CO_2_ and alveolar dead space stayed lower but differed to a lesser (non-significant) extent. Charron et al. [46] described similar changes of arterial CO_2_ and dead-space variables in adult ARDS patients after a proning manoeuvre. In the mentioned study, PP induced a decrease in PaCO_2_ and Vdalv/Vte (with the maximum 6 to 9 h after the manoeuvre) and an increase in compliance which was related to an improvement in respiratory mechanics. An experimental PET study by Richard et al. proved that the combination of PP and PEEP (PEEP 10) had the most impressive effect on a homogenous ventilation distribution along the dependent-to-non-dependent dimension in lung-injured pigs [47]. Therefore, our study not only confirmed the immediate positive effect of the proning manoeuvre but also documented successful pulmonary recruitment by significant higher PaO_2_/FiO_2_ and significant lower dead-space values (Vdal and Vdphys) in the prone group after the decremental PEEP phase (Figure 2).

According to our EIT data, ventilation distribution is homogenous at baseline and changes to be predominant in the non-dependent thus dorsal regions after the proning manoeuvre. Thereafter, the combination with the incremental PEEP leads to a homogeneous distribution of ventilation between dependent and non-dependent regions (Figure 3B). Similar findings have been described by Meier et al. [16] in an EIT study with an animal model in the supine position only. Our animal data conform to Guerin et al. showing in humans that a protective and open lung PEEP has an additional effect on oxygenation and ventilation parameters in the prone position [39].

The results of our study reflect pulmonary derecruitment with increased shunt fraction during ALI and mirror lung overinflation at the maximum PEEP of 30. After induction of ALI, alveolar collapse and increased pulmonary shunt lead to reduced oxygenation, reduced compliance and increased alveolar dead-space fraction. The increased arterio-end-tidal CO_2_ difference is even more pronounced in the supine group. The shunt fraction of the prone group seems to stay lower within the whole trial. Whereas parameters of oxygenation (PaO_2_/FiO_2_) and ventilation (PaCO_2_, etCO_2_) reflect global pulmonary shunt, they may not give information on pulmonary overdistension during the PEEP trial. During the phase of maximal airway pressure at PEEP 30, Cdyn is reduced and dead-space variables are increased in both groups. This reaction reflects overdistension of pulmonary parenchyma. However, lowest alveolar dead space (Vdalv/Vte) during the PEEP trial for each group is documented at decremental PEEP 20d reflecting the reduction of shunt after recruitment of injured lung parenchyma. Richter et al. found in a surfactant-deficient model of lung injury in sheep that PP improved gas exchange by restoring aeration and decreasing shunt. Perfusion in dorsal lung regions was preserved, and the distribution of ventilation was more uniform [32]. Similar findings were also described by Maisch et al. in adults with healthy lungs undergoing general anaesthesia after a recruitment manoeuvre and PEEP titration. The highest compliance value together with the lowest alveolar dead space (at PEEP 20d) indicated optimal alveolar recruitment whereas functional residual capacity and PaO_2_ were insensitive to document alveolar overdistension [30].

Theoretical limitations of the study are a) documentation of pulmonary shunt by means of pulmonary catheterization was not implemented in the study design, b) fundamental differences of tetrapod vertebrates compared to humans regarding prone or supine position in lung injury models have not been described in literature.

## Conclusions

Our study confirms the well-established positive effect of prone position on oxygenation and ventilation in acute lung injury. EIT documented a homogenous distribution of ventilation in dependent and non-dependent lung regions in the prone position during the PEEP titration. Non-invasive methods at the bedside are feasible to determine the optimal individual PEEP for alveolar recruitment while avoiding lung overdistension. Our study could demonstrate that alveolar dead space reflects pulmonary shunt, caused either by atelectasis or by over-inflation. Acquisition of alveolar dead space by means of volumetric capnography and registration of dynamic compliance together with the visualization of ventilation by means of EIT may help to define the optimal PEEP in acute lung injury.
